# Multidisciplinary Approach for Adult Patients With Childhood-Onset Chronic Disease Focusing on Promoting Pediatric to Adult Healthcare Transition Interventions: An Updated Systematic Review

**DOI:** 10.3389/fped.2022.919865

**Published:** 2022-06-14

**Authors:** Rie Wakimizu, Keita Sasaki, Mitsuki Yoshimoto, Akari Miyazaki, Yumiko Saito

**Affiliations:** ^1^Department of Child Health Care Nursing, Division of Health Innovation and Nursing, Faculty of Medicine, University of Tsukuba, Tsukuba, Japan; ^2^Master Program in Nursing Science, Graduate School of Comprehensive Human Sciences, University of Tsukuba, Tsukuba, Japan; ^3^Doctoral Program in Nursing Science, Graduate School of Comprehensive Human Sciences, University of Tsukuba, Tsukuba, Japan

**Keywords:** childhood-onset chronic disease, systematic review, healthcare transition interventions, multidisciplinary approach, adolescents

## Abstract

**Introduction:**

Owing to improved prognosis, the number of adult patients with childhood-onset chronic disease (APCCD) has increased. In this systematic review, we evaluated a multidisciplinary approach toward APCCD, focusing on promoting pediatric to adult healthcare transition interventions and their effects.

**Methods:**

We reviewed literature comparing the effects of pediatric to adult healthcare transition interventions in children and adolescents with childhood-onset chronic disease, using PubMed, MEDLINE, and CINAHL, from 2010 to 2021 (keywords: “transition,” “children,” “intervention,” “healthcare,” etc.). The inclusion criteria were as follows: (i) original studies, (ii) studies on pediatric to adult healthcare transition interventions in children with chronic disease, (iii) patients including “adolescents” aged 12 and older receiving intervention, and (iv) studies that included the four elements of the PICO model: Patient/ Problem, Intervention, Comparison and Outcome model.

**Results:**

After evaluating 678 studies, 16 were selected, comprising topics such as “individual education programs” (*n* = 6), “group meetings” (*n* = 6), “active learning using information and communications technology” (*n* = 2), and “transition clinics” (*n* = 2). The effects obtained varied, depending on the contents and methods of the intervention. Additionally, there was no evidence of adverse outcomes from these interventions.

**Conclusions:**

Pediatric to adult healthcare transition interventions provide systematic support for the transition, patient independence, and social participation; thus, they should be adopted based on their expected effects.

## Introduction

Due to the rapid progress in the field of pediatric medicine, including fetal medicine and neonatal care, in recent years, the infant mortality rate per 1,000 births in Japan has decreased from 3.2 (2000) to 1.8 (2020), alongside the neonatal mortality rate reducing from 1.8 (2000) to 0.8 (2020); this trend shows that many lives have been saved during the perinatal period (1). Such advances in medical care have led to a reduction in mortality from childhood-onset diseases (2), and a subsequent increase in the number of adult patients with childhood-onset chronic disease (APCCD). Along with this, support for the transition from pediatric to adult medical care is gaining importance. The transition period to adulthood is one in which physical and psychosocial maturity progresses rapidly even in the developmental stage, alongside new problems due to physical maturity, changes in social roles, maturation of the patient's personality, etc. Therefore, it is necessary to provide seamless medical care in pediatric and adult care.

The transition to adult care is defined as “a purposeful and systematic transition from pediatric to adult care, including the process responsible for managing one's own chronic illness” (3, 4). Self-management for transition includes not only management of illness symptoms but also that of medication and consultation appointment, and communication with healthcare professionals (5). Furthermore, studies investigating the transitional experiences and needs of adolescents with chronic illness have identified a variety of needs. These comprise the importance of peer support; future and occupational issues; pathology and insurance and related information; medication (type, dosage, and side effects); disease knowledge, including understanding of genetic predisposition; difficulty in interacting with the doctor; changes in the doctor-patient relationship due to growth and development; taking responsibility for one's own health; and not relying on parents for disease management (6, 7). Therefore, it is necessary to build comprehensive support for various factors regarding the transition of APCCD to adult medical care.

Even in Japan, interest in adult transitional medical support has been high. In 2015, the Ministry of Health, Labor and Welfare developed tools for transitional support and the “Children with Chronic Specific Diseases Child Transitional Medical Support Model Project” (8), the purpose of which was to improve the transitional medical system by holding training at the pertinent medical institutions. The transitional medical support in this project implied “support for the process of transitioning from pediatrics to adult-centered medical care.” Its goal was to “provide independence support (autonomous support) to encourage patients to acquire self-care skills and actively participate in decision-making, and to lead to appropriate care in adulthood without interrupting necessary care.” Although various adult transition programs aimed at transitional medical support are being developed and evaluated abroad, there are currently no studies that integrate the adult transition programs and their results to evaluate the effectiveness of these interventions.

The PICO (Patient/Problem, Intervention, Comparison, and Outcome) of this study setting was as follows: “Does the adult healthcare transition program for APCCD proceed transition effectively compared with no intervention.” Along with the systematic review, it aimed to integrate the transition support program for the medical support of APCCD and its intervention effect in Japan and overseas, evaluating its effectiveness for adult transition in a multidisciplinary manner.

## Methods

### Search for Studies

The search date was September 24, 2021, and PubMed, MEDLINE and Cumulated Index to Nursing and Allied Health Literature (CINAHL) were used as the databases. The search target period was set from 2010 to 2021 in each database. Studies were extracted from January 2010 to May 2021 for PubMed, and from January 2011 to December 2020 for both MEDLINE and CINAHL.

### Search Keywords for the Papers

The search keywords were a combination of “transition/transfer,” “care/health care/treatment/therapy,” “children/young adult/young people/pediatric,” “intervention,” “chronic disease/chronic illness/APCCD/disease,” and “self-management/selfcare/readiness.”

### Inclusion and Exclusion Criteria

Inclusion criteria for this systematic review were as follows: (i) being an original paper; (ii) being a paper on interventions related to adult transition of children with chronic diseases; (iii) including “adolescents,” with the intervention target being individuals aged 12 and older; and (iv) papers with a comparison of the results before and after the intervention being shown. Exclusion criteria for the papers were as follows: (i) the participant had difficulty in self-care due to severely handicapped children, chromosomal abnormalities, intellectual disabilities, etc.; (ii) the paper was intended for palliative care; (iii) the fact-finding survey did not involve intervention; and (iv) the intervention included participants under the target age.

### Selection of Target Studies

Excluding duplicate studies from the ones obtained through each database, we perused the titles and abstracts and excluded those that did not fall under P and I of the PICO in this systematic review. We carefully read the entire contents of the papers after the exclusion, eliminated those that lacked pre- and post-comparisons of the intervention, and finally examined the remaining papers. All the authors worked in consultation during each process.

### Bias Risk Assessment

The Risk Of Bias In Non-randomized Studies – of Interventions (ROBINS-I) assessment tool (Cochrane | Trusted evidence. Informed decisions. Better health.) was used for the bias risk assessment of the target studies. The tool was capable of measuring confounding, selection, measurement, and missing data, as well as reporting bias, and was ranked low, moderate, serious, no information (NI), and not applicable (NA). The evaluation was conducted independently by the researchers, and the results were shared and discussed among them. Further discussions were held on the suspicions that emerged as a result, and consensus was reached.

### Data Extraction Method

From the target papers, “references,” “study design,” “interventions,” “person or group providing interventions,” “participants,” “outcomes,” “main results,” “limits of the study,” and “overall risk of bias” were entered in a table prepared in advance, and made into a list. The data were extracted independently by the researchers, and the results were shared and discussed among them. Based on the opinions obtained through the discussion, the extracted contents were added and revised, and a list was created.

## Results

### Breakdown of Analysis Target

**Figure 1:**
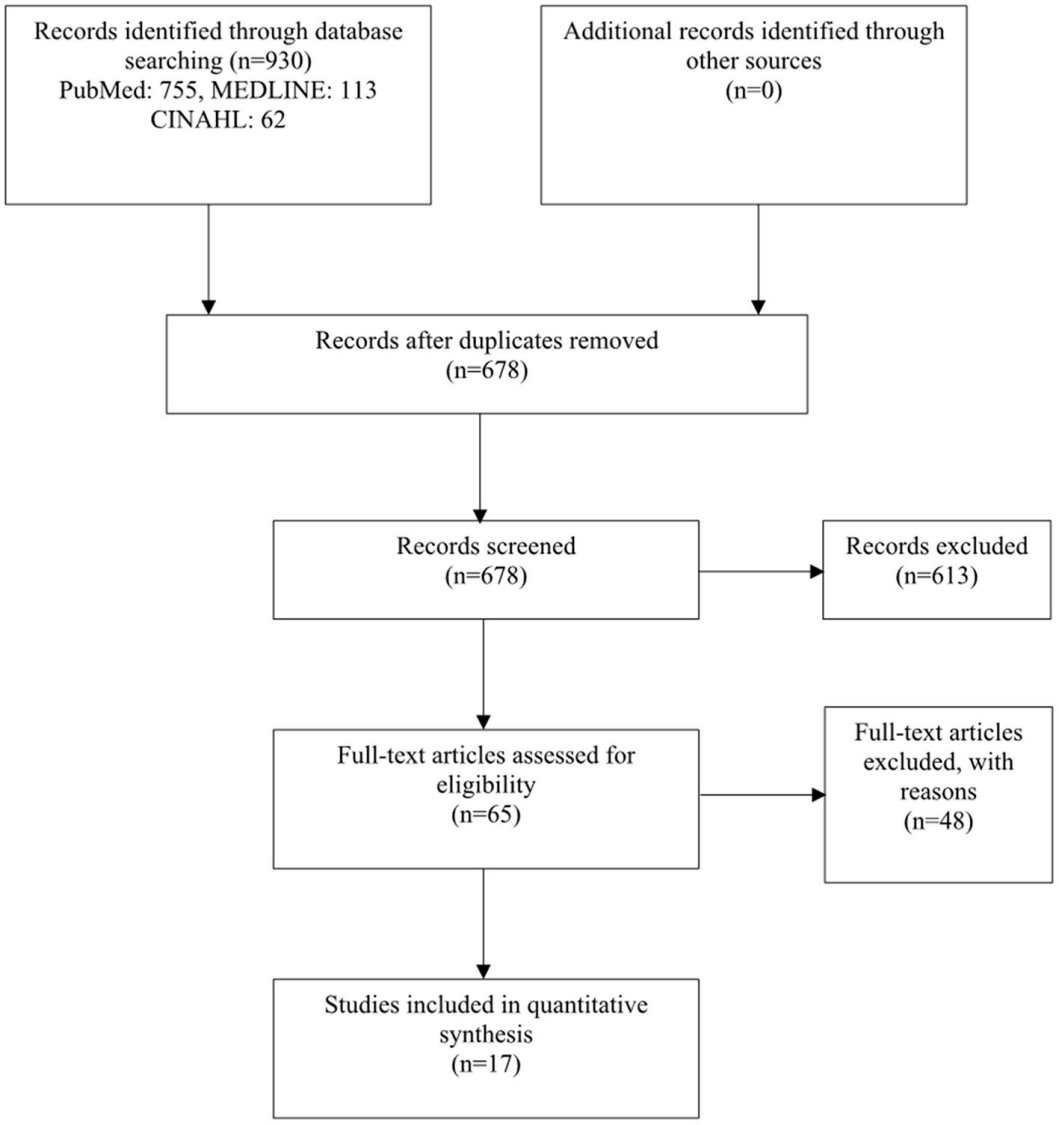
Flowchart of the study selection.

Of a total of 678 cases obtained from the keywords, the abstract and text were carefully read. And we selected 17 studies, but one of the studies actually did not meet the PICOs. Because of that, we finally analyzed 16 studies. Figure 1 shows the process of selecting the target papers.

### Research Report Year

**Figure 2:**
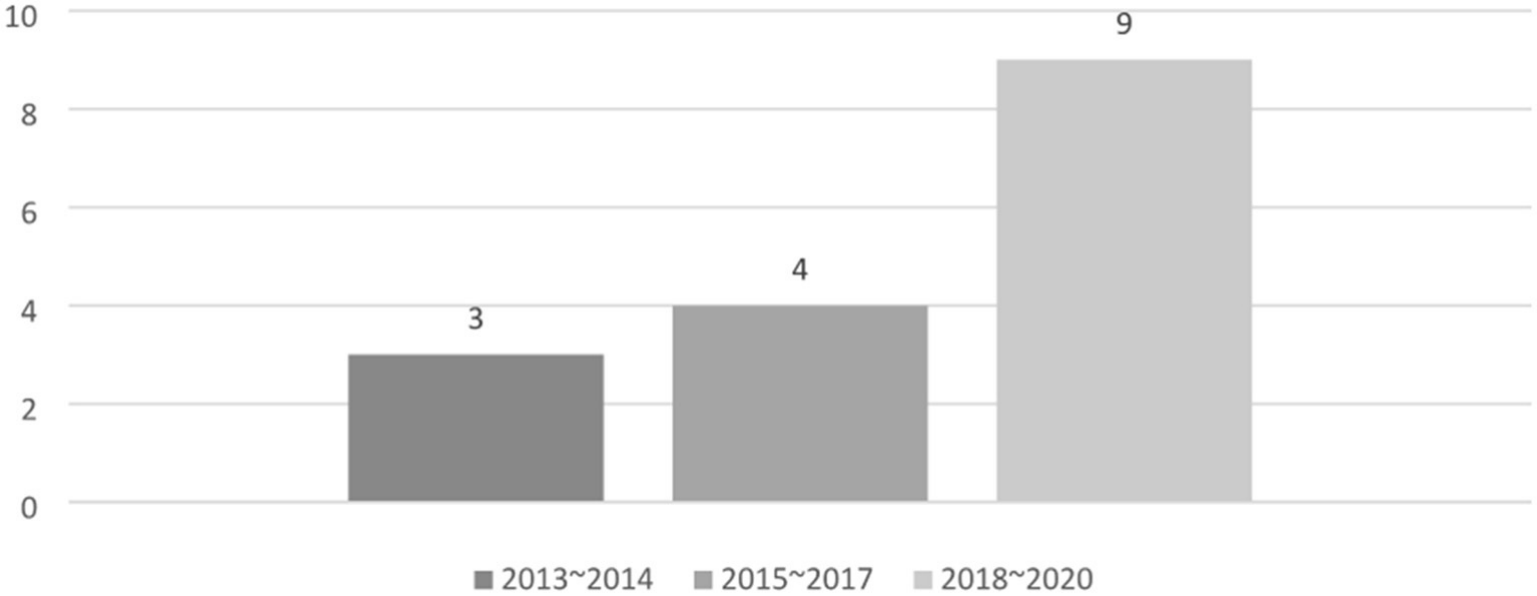
Number of papers by year.

When the 16 papers considered were classified by their reporting years, one study in 2013 (9), two in 2014 (10, 11), one in 2015 (12), one in 2016 (13), two in 2017 (14, 15), four in 2018 (16–19), two in 2019 (20, 21), and three in 2020 (22–24) were obtained. Of the 16 studies, nine had been reported since 2018, accounting for more than half of the studies considered (Figure 2).

### Participants

Most of the studies targeted were regarding the late teens, as they were in the process of transitioning from childhood to adulthood. Of the 16 studies, the minimum age of the target population was 11 years, and the maximum was 25 years.

**Figure 3:**
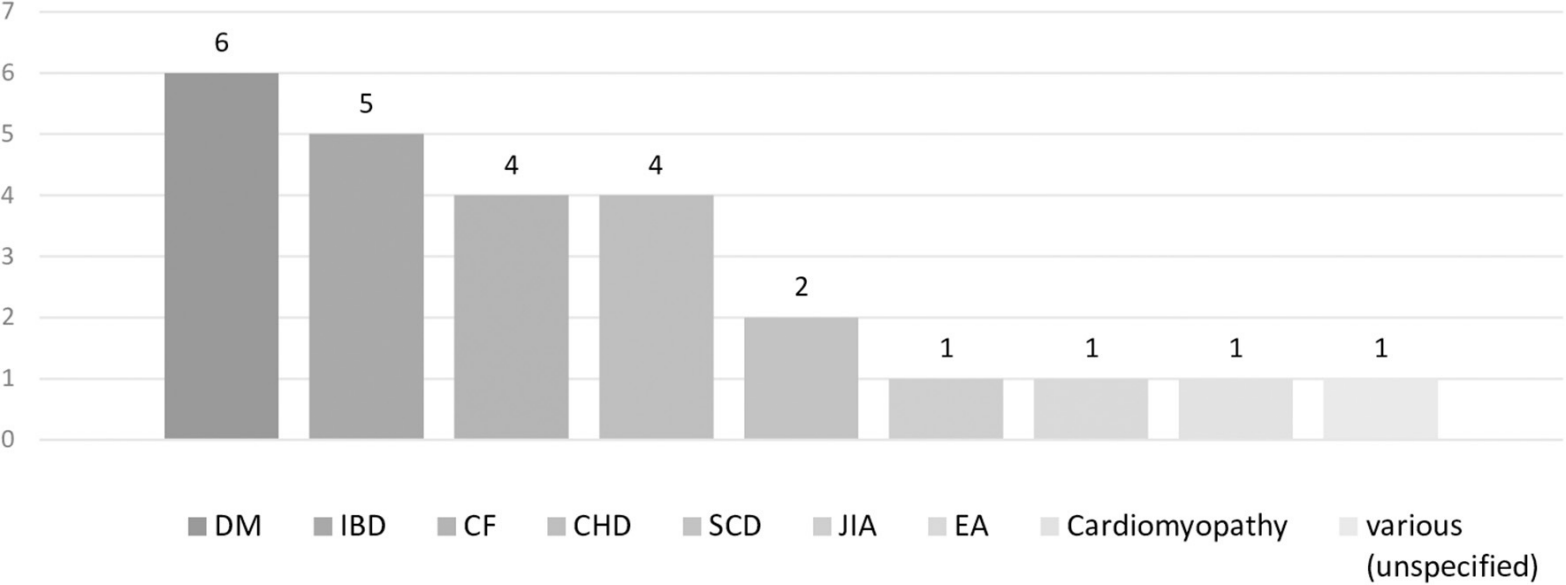
Number of papers by disease of the participant.

The breakdown of the diseases examined was shown. Diabetes mellitus (DM) was included in six papers, followed by inflammatory bowel disease (IBD) in five, cystic fibrosis (CF) in four, congenital heart disease (CHD) in four, sickle cell disease (SCD) in two, juvenile idiopathic arthritis (JIA) in one, esophageal atresia (EA) in one, and cardiomyopathy in one. There were six studies involving patients with 2–3 diseases rather than a single disease. Additionally, there was one study on patients with common chronic illnesses, involving 1–6 patients for 15 illnesses (Figure 3).

### Study Design

As a result of classifying the literature by research design, there were six quasi-experimental studies of one-group pre- and post-test design (9, 12, 17, 19–21), seven studies of randomized controlled trial (RCT) research (10, 11, 13, 16, 18, 22, 23), and three non-RCT studies (14, 15, 24). Since the pure experimental study of one-group pre- and post-test design was not a controlled study, it was difficult to determine to what extent interventions and other factors contributed to self-management, transitional readiness, and improved disease activity. In the non-RCT studies, the intervention and control groups were assigned based on the patients' wishes. Therefore, the possibility of selection bias due to the large number of patients or their families who were more interested in the transition from childhood to adulthood could not be ruled out in the intervention group.

### Bias Risk Assessment

Bias risk assessment was performed using ROBINS-I for the target studies; one study (11) was assessed to be low, 14 (9, 10, 12–23) were assessed to be moderate, and one (24) was assessed to be serious. The latter was more likely to be biased due to loss of data.

### Content of Intervention

**Figure 4:**
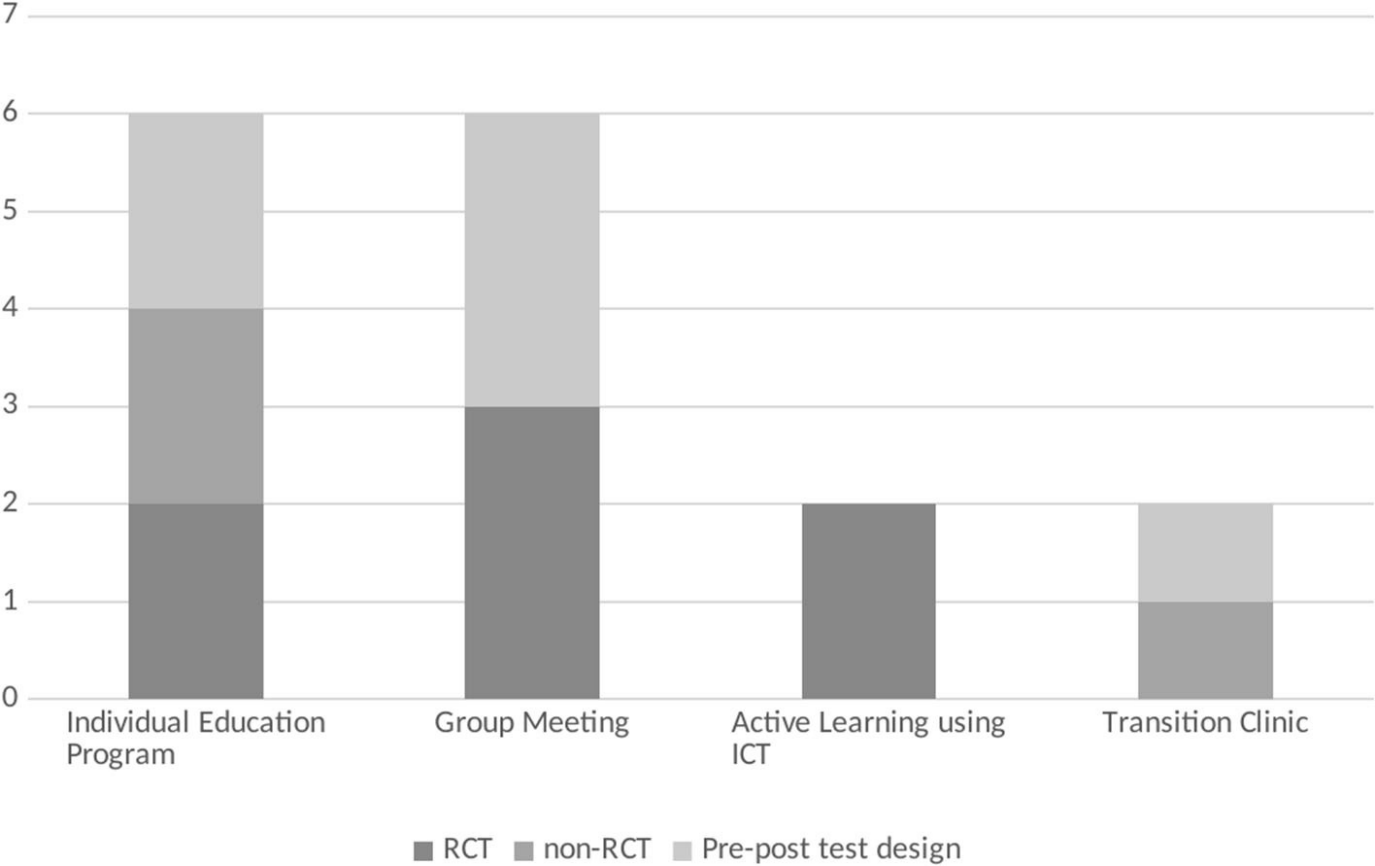
Number of papers by the content of the intervention.

The 16 studies analyzed were roughly classified based on the type of intervention. They were classified into four categories: Individual Education Program (IEP) (10, 14–17, 20), Group Meeting (GM) (9, 13, 18, 19, 21, 22), Active Learning using Information and Communications Technology (ALICT) (11, 23), and Transition Clinic (TC) (12, 24) (Figure 4). Educational interventions for coping with the disease and self-management were the actions common to all four groups. From the aspect of patient's age, the characteristics of how the programs were used according to the different ages were not mentioned in any of the studies.

### More Specific and Detailed Intervention Content and Its Effects

For each type of intervention, we showed the specific and detailed content of each study and their effects (Table 1).

**Table 1:** List of study design and intervention contents of review papers.

**Content of interventions**	**References**	**Study design**	**Interventions**	**Person or group providing interventions (supporter)**	**Participants**	**Outcomes**	**Main results**	**Limits of the study**	**Overall risk of bias**
IEP	Mackie et al. (16)	RCT	A one-on-one session between the nurse and the patient will be held on the appointment date at the clinic and 2 months later (two times in total for 1 h each). 1st: Creating a My Health passport (CHD anatomy, treatment history, name/amount/efficacy of medications taken, future complications, name and location of local adult cardiovascular doctor, introduction of health care website for young people, case study on alcohol intake, introduction to adult CHD clinic consultation, setting of one education-related goal, etc.) 2nd: Confirmation of goals set in Part 1, watching / discussing short videos on communication with medical professionals, role-playing, related materials/website introduction In both sessions, the contents will be sent from the nurse by email etc. within seven days after the implementation. For all patients, including the control group, the pediatrics sent medical records to the adult clinic, and the adult clinic sent notifications about the date of first visit to the patients.	Pediatric clinic nurses	121 patients with Moderate or complex CHD, 16–17 years old (58 in the intervention group and 63 in the control group)	Excess time between pediatric and adult CHD treatment: (time interval from the final pediatric visit to the first adult visit)-(recommended time interval for these visits) My Heart Scale TRAQ Williams' self-management scale Assessment of self-management by cardiologist Incidence of cardiac reintervention surgery or catheterization 12 or 24 months after enrollment	At the recommended time of initial booking for adults (excess time = 0), intervention participants were 1.8 times more likely to book within a month (95% CI: 1.1 to 2.9; Cox regression, *p* = 0.018). Intervention participants are 3.0 times more likely to make an appointment within a month when the overtime is 6 months (95% confidence interval: 1.1–8.3). The intervention group had higher scores at 1, 6, 12, and 18 months on the My Heart knowledge survey (mixed models, *p* <0.001) and TRAQ self-management index (mixed models, *p* = 0.032).	Participants may have had a participant bias of relatively high (or low) knowledge and self-management ability compared to the general population with CHD. It is a nurse-led intervention and variability among nurses is inevitable. Although the grouping is not known to cardiologists and other clinic staff, it is possible that the grouping may be mistakenly known.	Moderate
IEP	Mackie et al. (10)	RCT	About the transition and its importance, Creating a My Health passport (name of heart disease, treatment history, name and purpose of the drug being taken, whether or not there is a need to prevent endocarditis), Confirmation of cardiac anatomy, About three possible heart complications in the future, Confirmation of contact and location of local cardiologist, Distribution of related websites and materials (sexually transmitted diseases and substance abuse), Case study of alcohol intake, etc., Follow-up recommended by nurses by email etc. Control group: Cardiologists or nurses at cardiovascular clinics promote self-management and communication skills and provide non-standardized information about the heart, either verbally or in writing.	One experienced cardiovascular nurse	58 patients with Moderate or complex CHD or cardiomyopathy, 15–17 years old [(25) in the intervention group and 31 in the control group]	Evaluation of self-management (1) and self-advocacy (2) TRAQ [e.g., (1) filling out prescriptions, making appointments for consultations, (2) communication skills and use of school and local resources] Confirmation of knowledge about heart condition (a unique scale of the study): My Heart Scale (evaluated by a pediatric cardiologist, a cardiologist, a research assistant)	Compare the intervention group with the regular care group at 6 months after the intervention. The average self-managed TRAQ score is 3.59 (± 0.83) vs. 3.16 (± 1.05) (*p* = 0.048, adjusted by baseline score), respectively. The average self-advocacy TRAQ score is 4.38 (±0.56) vs. 4.01 (±0.95) (*p* = 0.18). The average My Heart score is 75% (±15) vs. 61% (±25) (*p* = 0.019).	Inevitable variability among intervention nurses Self-management evaluation is self-reporting Long-term follow-up and intervention at an adult heart clinic should not be evaluated.	Moderate
IEP	Ladouceur et al. (14)	Non-RCT (Allocation based on experience of participating in educational programs)	Individual consultation by a specialist nurse at the time of regular consultation in cardiology Discussions on potential cardiac symptoms requiring medical assistance, the importance of long-term and frequent consultations by cardiologists, dangerous behaviors to avoid, health habits and prevention of sexually transmitted diseases, and Osler	Two experienced cardiology nurses	115 adolescents and young adults with CHD 14–19 years old (22 in the intervention group and 93 in the control group)	Questionnaire with 29 items for boys and 34 items for girls regarding six areas (Knowledge about CHD and heart surgery, heart follow-up and treatment, heart symptoms and self-management, dangerous behavior and healthy lifestyle, insurance and professionalism, recurrence, and	The average value of the overall health knowledge score (out of 20 points) in the education group was significantly higher than that in the comparison group (11.7 ± 3.5 vs. 8.6 ± 3.2; *P* <0.001).	The number of intervention groups is small. Possibility of selection bias because it is not an RCT.	Moderate
			endocarditis, etc. Standard care was provided to all participants, including the control group. Some participants were provided with oral and / or written information at the discretion of the cardiologist.			gynecological problems)			
IEP	Dingemann et al. (15)	Non-RCT (Assign to desired group)	Transition-specific patient/parent education program ModuS-T General purpose module: Consider changes in adulthood, consideration of changes to the attending physician, health system, career development, social connections, stress coping, and specific changes in daily life Symptom Modules: Family Planning, Alcohol, Drugs 12 units 45 min each (2 days) Control group: Normal care (no details)	General-purpose module: 1 psychologist each Symptom Module: Experienced Pediatric Surgeon	Patients with a history of EA and their parents, 14–21 years old (10 in the intervention group and 19 in the control group)	Program satisfaction: ZUF-8 Confirmation of knowledge in the program: transition-specific knowledge questionnaires HRQOL:DISABKIDS Chronic Generic Measure-37 Adolescents' own health care efforts: German version of the Patient Activation Measure-13D (PAM-13D)	The patient's transitional knowledge was 36% correct before the program, but improved 18% immediately after the intervention, with a 56% correct answer rate (*p* = 0.004). However, there was no change in the control group (54 vs. 52%, n.s.). Parental transitional knowledge did not change after the intervention (correct answer rate 66:67%, ns). There was no detectable effect on HRQOL (intervention group 79.7 vs. control 81.5, DISABKIDS general score) and self-management (intervention group 44.4 vs. control 41.4, PAM-13).	The number of research participants is small Three evaluations were performed before the intervention, immediately after the intervention, and 4 weeks after the intervention, but the follow-up period was short. Since it is not an RCT, the participation rate of those who are interested in research is high.	Moderate
IEP	Skov et al. (17)	Quasi-experimental study of group one pre-post-test design	Divided medical care for parents and children _Consultation by nurses focusing on life, self-management, psychosocial risk, and resilience (twice a year) Annual parental event (interaction with CF Center, Adolescent Center staff, etc.)	Doctors and nurses	40 CF patients, 12–18 years old	Preparation checklist (created by researchers) Quality of life related to the health of CF patients: CF Questionnaire Revised teen/adult version (CFQ-R) Height, weight, body mass index, lung function [forced vital capacity (FVC) and forced expiratory volume in 1 second (FEV1)]	Preparedness checklist scores increased significantly from an average of 64 points to 76 points at baseline and at 12-month follow-up (*p* = 0.002). However, the number of respondents who answered two points was *n* = 15. In QOL, “role restriction,” which indicates the ability to respond to daily life, increased significantly (*p* = 0.046), and “respiratory symptoms” decreased (*p* = 0.003). Lung function was flat.	No one dropped out of the program, but some participants were too busy to complete the checklist.	Moderate
IEP	Gray et al. (20)	Quasi-experimental study of group one pre-posttest design	Transition coordinator at regular visits provides 15–20 min interviews with patients and family, customized education on self-management and parent-to-patient responsibility transfer (once a year) _Follow-up of goals set by phone or email (within 3 months after interview).	Transition coordinators (social workers)	135 IBD adolescents and young adults and their families, 14–17 years old	Evaluation of self-management skills for health: TRAQ Clinical disease activity: PGA (Physician Global Assessment) Evaluation before (T1) and 1 year after (T2) the transition coordinator's intervention	The TRAQ score was 68.13 points before the intervention, but increased to 74.38 points after the intervention, showing a significant increase in transition readiness and self-management ability. Of the 20 items of self-management ability acquired, it was 7.07 before the intervention, but increased to 8.20 after the intervention, showing a significant increase.	Because it is not a controlled study, it is not possible to determine to what extent the transition coordinator and other factors contributed to self-management, transition readiness, and improvement in disease activity. Self-management evaluation is self-reporting.	Moderate
GM	Markwart et al. (22)	RCT	Educational program consisting of nine modules (transition to adult medical institutions, future plans and occupations, communication about illness with peers and parents, etc.) The control group does not participate in the intervention, but answers the questionnaire at the same time as the intervention group.	Psychologists and pediatricians, adult medicine doctors, young adults with the same chronic illness as intervention participants	723 patients with chronic disease (T1DM, CF, IBD) Average 16.98 years old, SD = 1.64 (407 in the intervention group and 316 in the control group)	General Self-Efficacy scale (GSE) Transition Competence Scale (TCS) PAM	The intervention group had significantly higher patient empowerment (PE) scores than the control group.		Moderate
GM	Schmidt et al. (18)	RCT	Educational program consisting of nine modules (transition to adult medical institutions, future plans and occupations, communication about illness with peers and parents, etc.) _The control group received routine care, including medical care based on individual needs and counseling as needed.	Transition Workshop: Psychologist, Pediatrician. Some modules: Young adults with chronic illness and adult medical professionals.	285 IBD and DM patients. Average 16.57 years old, SD = 1.31 (125 in the intervention group, 24 in the dropout, 117 in the control group, 19 in the dropout)	Health-related TCS The German short version of the DISABKIDS Chronic Generic Measure The EUROHIS QOL-8	The intervention significantly improved transitional ability in both groups, but higher in IBD patients.		Moderate
GM	Schmidt et al. (13)	RCT	Educational program consisting of eight modules (transition to adult medical institutions, medical system, future plans and occupations, parting with parents, communication about illness with peers and parents, stress management, resource activation, etc.) _The control group received routine care, including medical care based on individual needs and counseling as needed.	Psychologist, pediatricians. In some modules, young adults with chronic illnesses and doctors in the adult field.	274 adolescents with T1DM, CF and IBD, 13–22 years old. (142 in the intervention group and 132 in the control group)	Health-related TCS GSE PAM13-D Satisfaction with health care (CHS-SUN self) The German version of the EUROHIS QOL-8 A short form of WHOQOL-Bref The German short version of the DISABKIDS Chronic Generic Measure	The intervention group had a significant effect on transition ability, self-efficacy, and satisfaction with school care 6 months after the intervention, but not on patient activation and quality of life.	The failure to show changes in quality of life may be due to the fact that the measurement range is too wide for general QOL measurement methods.	Moderate
GM	Maslow et al. (9)	Quasi-experimental study of group one pre-post-test design	Discuss diagnosis, how to deal with illness, how to interact with doctors, school problems, friendships, family relationships, etc. while interacting with high school patients and university mentors at a dinner party (2.5 h a month).	Mentors (university students under the guidance of specialized groups), program directors, volunteer staff (pediatrics, psychiatric residents, group discussion facilitators, child life therapists, medical students, etc.)	20 patients with chronic disease Average 15.4 years, SD = 0.3 years	Connection with society: the University of California Los Angeles (UCLA) Loneliness Scale Competence and confidence: TRAQ Questionnaire to program graduates (high school student participants/university student mentors): Current and past educational experience (high school graduation, degree acquisition, current academic status, etc.)	Loneliness decreased from 46 to 38.5 (*p* <0.001), and health care self-assertion increased from 3.8 to 4.2 (*p* <0.001).	It is not an RCT and there is no control group setting.	Moderate
GM	Johnson et al. (21)	Quasi-experimental study of group one pre-posttest design	Program in line with transition readiness assessments, prescription medications, guidelines to prevent a pain crisis, and personal care and health care guidelines. Watch video for SCD patients in their teens. Q & A session with four questions of “Incorporating Health Care Transition Services Into Preventive Care for Adolescents and Young Adults: A Toolkit for Clinicians.” [(1) Reassurance for your own health management, (2) Timing when primary care or medical examination by a specialist is required, (3) The importance of self-health management such as filling out prescriptions, taking independent medicine,	No information	10 patients from SCD Pediatric Hematology Clinic. * No details about the age of the target person. * Inclusion criteria 18 years old and over	TRAQ	The total average score for women improved from 3.46 to 4.31, while for men it improved slightly from 3.19 to 3.28 before and after the intervention. For men, there was a slight improvement after the intervention in drug management, schedule management, and understanding of health problems. For women, improved medication management, health problem tracking, medical conversations, and daily life management.	Further investigation by RCTs is needed to address the limitations of the study.	Moderate
			carrying insurance cards, and making appointments for consultations, (4) Questions and concerns about changing to an adult clinician].						
GM	Essaddam et al. (19)	Quasi-experimental study of group one pre-posttest design	2–3 h/time group (8–12 people) meetings for children and their families. Introducing and asking questions about the adult team. Distribution of “diabetes health passport.”	Pediatric team (doctors, nurses, nutritionists) and adult teams (doctors, nurses, nutritionists, secretaries).	48 T1DM patients, 14.5–23.2 years old	HbA1c Number of hospital visits Number of hospitalizations	The HbA1c value decreased significantly 1 year after the transition to adulthood, with an average decrease rate of 0.93 ± 1.69%. The number of people who achieved HbA1c <7.5% increased to 8%.	It is possible that the group is originally highly motivated because it is a person who wishes to participate in an outpatient consultation / program for more than 2 years.	Moderate
ALICT	Huang et al. (11)	RCT	Disease management with MD2Me (eight months). Disease management and communication skills on the website, lifestyle education, case studies by disease (2 months). Short message and question delivery to confirm comprehension (3–5 times/week). 2 months later, disease management and information and weekly reminder messages on the website. Providing an automated SMS algorithm to support disease management decisions and a communication portal with the medical team. Control group: Receive monthly messages about common health issues by mail or email.	Medical team	81 adolescents (ACD) with IBD, CF and T1DM, 12–20 years old. (40 in the intervention group and 41 in the control group)	TRAQ PAM Frequency of patient-led communication. Disease status The Karnofsky Performance Scale, a functional status assessment scale Pediatric Quality of Life Scale (PedsQL) The Test of Functional Health Literacy in Adults	MD2Me participants showed significant improvements in disease self-management, health-related self-efficacy, and patient-led communication at baseline, 2 and 8 months, compared to the control group (*p* = 0.02, *P* = 0.02, *p* <0.001).	The sample size is relatively small, it is a single facility, and there is a difference in intervention frequency between the intervention group (once a week) and the control group (once a month).	Low
ALICT	Crosby et al. (23)	RCT	Group session (6 weeks), interpersonal booster session. Symptom recording by companion app (iManage). Control group: Education on SCD and general health was conducted by telephone for 6 weeks.	Doctor, two research facilitators (psychologist, psychology fellow, or graduate student in psychology)	53 AYA patients with SCD, 13–21 years old. (26 in the intervention group and 27 in the control group)	Self-efficacy: PAM-13 Self-management skills: TRAQ-5, UNC TR_X_ANSITION Scale SCD Knowledge: 25 disease-specific knowledge questionnaires Health Motivation: the Treatment Self-Regulation Questionnaire (TSRQ) Health-related quality of life HRQOL: the PedsQL sickle cell disease Module	In the intervention group, there was a significant improvement in self-efficacy (8-point change), with Moderate effect sizes, *P* = 0.09 and η^2^ = 0.06. There was also a statistically significant improvement in self-management skills (tracking health), *P* = 0.04.	The sample size is small.	Moderate
TC	Gaydos et al. (24)	Non-RCT The control group was selected from patients who had visited the Pediatric Cardiology Clinic before the transition clinic was opened.	Development of Subspecialty Pediatric Heart Clinic in January 2016 (once a month). Individual education is provided at the transition clinic. First visit: Explanation of transition method and importance, confirmation of understanding of one's disease, introduction of adult cardiovascular clinic, self-management goal setting, interview with adult CHD nurse. Second and subsequent visits: Re-education regarding heart disease, resetting self-management goals, interviews with adult CHD nurses, etc. The control group was a CHD patient who visited the Pediatric Cardiovascular Clinic for 3 months (December 2015–February 2016) and received normal medical care.	Pediatric cardiologists, adult CHD nurses	54 patients who visited the Pediatric CHD transition clinic, 16–21 years old. (54 in the intervention group and 53 in the control group)	participant “lost to follow-up” rate TRAQ PedsQL 3.0 Cardiac Module	The percentage of “unfollow-up” that improved follow-up rates in adolescents and young adults was 7.3%, significantly lower than in the control group (25.9%, *p* <0.01). There was no significant difference in the TRAQ score in the follow-up score.	Selection bias is likely to occur because referral by a cardiologist was required.	Serious
TC	Staa et al. (12)	Quasi-experimental study of group one pre-posttest design	Transition Clinic/Youth Clinic establishment and transition coordinator, transition protocol setting, regular consultation between pediatric and adult care (including multidisciplinary transition meetings). _Individual Transition Plans Personal consultation Providing information leaflets and websites for young people.	Pediatric and adult medical team	AYA generation patients with T1DM or JIA (T0) At the start, 389 people, 11 to 25 years old (T1) 1 year later (at the time of completion), 430 people (T2) 2 years later, 207 people	The Independent Behaviors During Consultations Scale (IBDCS) General independence during consultations Topics Discussed During Consultations Scale (TDDCS) The Dutch version of GSE On Your Own Feet Self-Efficacy Scale: OYOF-SES)	IBDCS, General independence during consultations, and GSE increased significantly from T0 to T2, and Your Own Feet Self-Efficacy Scale also increased from T1 to T2 (all *p* <0.05–0.001).	There is a lack of information on how the intervention was actually performed and on clinical outcomes.	Moderate

#### Individual Education Program

Six studies involved individual programs, in which supporters provided direct face-to-face education, discussions, planning, consultations, follow-ups, etc., according to the requirements of individual patients. The contents of the individual programs were divided into five programs in which supporters provided one-on-one education to patients and their families, and one in which support was mainly provided for planning and implementation. In five education-centered programs, two were conducted as RCT study.

Of the education-centered programs, two RCT (10, 16) and one non-RCT research (14) were programs for CHD patients to gain understanding of diseases, confirmation of the current situation, points to be noted in future daily life, etc., through the creation of a My Health Passport developed in Canada. My Health Passport specifically included CHD anatomy, treatment history, names/amounts/benefits of medications taken, future complications, names and locations of local adult cardiologists, an introduction to a health care website for young people, case study (alcohol, smoking/street drug, sexuality/contraception), introduction to adult CHD clinic consultation, and setting of one education-related goal. The three studies centered on the My Health Passport, including an introduction to adult CHD clinics (16), education on sexually transmitted diseases and substance abuse (10, 14), setting goals for transition, and follow-up (10, 16).

All the three studies found that the intervention group displayed significantly higher knowledge scores for diseases and health than the non-intervention group. In two RCT studies (10, 16) that also included a program with transitional goals and follow-up by healthcare professionals at intervals, self-managed Transition Readiness Assessment Questionnaire (TRAQ) scores were higher than in the non-intervention group in both studies. In addition, compared to the non-intervention group, the transition time from pediatrics to adult specialty was shorter (16), and the self-assertion TRAQ score was higher (10).

A non-RCT study of patients with a history of EA (15) conducted the ModuS-T program developed for patients with type 1 diabetes mellitus (T1DM), IBD, and CF in Germany. Specifically, supporters held discussions with patients and their parents over a 2-day period regarding the changes occurring due to adulthood, examination of changes in the attending physician, health system, stress coping, among others. The study showed improved parental knowledge compared to the non-intervention group, but no significant effect on Health-Related Quality of Life (HRQOL) and self-management ability.

The transition program for patients with CF (17) in Denmark which is conducted as quasi-experimental studies of one-group pre- and post-test design, featured split care for children and parents to create an opportunity for the patient to be seen alone. In addition, two of the 12 consultations a year were held by nurses for 60-min consultations, providing opportunities for consultation on life, self-management, psychosocial risks, and resilience. An annual event for parents was also one characteristic of the program along with individual patient interventions. A mini-lecture on how to deal with chronic diseases and adherence by experts, discussions among parents, and a place to share experiences were provided. The study showed a significant reduction in readiness indicators and respiratory symptoms before and after the intervention.

The study targeting patients with IBD in the United States (20) which is also one-group pretest-posttest design research focused on the formulation and implementation of transition plans. Interviews with patients and their families were conducted by a transition coordinator (social worker) at the time of regular visits. Specifically, during a 15–20-min interview, the IBD Self-Management Handbook was used to explain the concept of transition, provide education on the transition of responsibility from parent to patient, and set migration goals for family and patients. Three months after the interview, a follow-up was conducted on telephone or email regarding the status of the goals set at the time of the interview. The study showed a significant increase in TRAQ transition readiness and self-management ability before and after the intervention.

#### Group Meeting

The number of studies that involved GMs to promote transition from childhood to adulthood through group education and discussions for patients and parents, was the same as that for IEPs. Among them, by participant, five studies were targeted at children who were patients, and one was targeted at both parents and children.

Of the five studies mainly for children, three RCT (13, 18, 22) were for patients with chronic diseases such as T1DM, CF and IBD in Germany. All programs were offered to four or more patients at a time for two consecutive days, including transition to adult care, health care systems, future plans and professions, illness communication with peers and parents, stress management, etc. The program was primarily conducted by psychologists and pediatricians, and incorporated group-work to facilitate knowledge and information exchange between participants. Of the three studies, in the one on patients with T1DM, CF and IBD (22), and the one on patients with DM and IBD (18), the intervention group had significantly higher empowerment and knowledge scores than the control group. In another study (13) of patients with T1DM, CF and IBD, it was reported that while the transition ability, self-efficacy and satisfaction with school care were significantly higher than in the control group, there was no significant effect on patient activation and quality of life.

Similarly, in a study (9) conducted as one-group pretest-posttest design research in the United States on patients with chronic illness, high school and university students with chronic illness interacted at a dinner party for about 2.5 h every month. After dinner, they were divided into small groups to select and discuss on various topics such as diagnosis, living with illness, and problems that occur at school. Before and after the program centered on the interaction of patients with the same illness, it was reported that the feeling of loneliness decreased and the self-assertion about health care improved.

In a group meeting (21) centered on patients with SCD, a session was held within the group on four questions regarding their own medical condition, self-management of medicines, reservations for medical institutions, etc., after watching a video created for teenage SCD patients transitioning from pediatric to adult care. From this one-group pretest-posttest design research, it was found that drug management, understanding of health problems, communication with medical staff, and improvement of health management in daily life were clarified.

GMs (19) for children with T1DM and their parents in North Africa were held once or twice a year in the pediatrics department of the hospital during school holidays. The group meeting lasted 2–3 h and was attended by 8–12 patients with their families and the hospital's pediatric team (two doctors, one nutritionist, and one nurse), and adult staff including adult endocrine doctors, diabetic nursing educators, nutritionists, and secretaries. Through group meetings, patients and their families had the opportunity to interact with new care providers and raise concerns and questions. Through the one-group pretest-posttest design research, HbA1c levels decreased significantly and more patients achieved HbA1c < 7.5% 1 year after the program intervention.

#### Active Learning Using ICT

Two programs were identified as ALICT to promote intervention. Both were RCT. There were three specific intervention methods: disease management and learning programs using ICT, and GMs.

Disease management using ICT was conducted in both studies, and it was recommended to provide and use tools for recording symptoms such as the applications “iManage” (23) and “MD2Me” (11). In particular, “iManage,” in addition to self-management such as progress report of self-management goals and input of daily pain and mood discomfort, had provisions for exchanging messages with other participants and sharing of a picture diary of weekly events.

Regarding the learning program (11), information on disease management, communication skills, and lifestyle was provided on the website for 2 months, and self-management in adulthood was learned through case studies by disease. A study (11) that combined ICT disease management and learning programs showed improvements in disease self-management ability, self-efficacy, and patient-led communication.

The ICT GM (23) was used in combination with the in-hospital face-to-face GM to hold a 90-min group session by Zoom. In a study (23) conducted in combination with ICT disease management and GMs, the intervention group had higher self-efficacy and self-management ability than the control group.

#### Transition Clinic

The TC for the adolescent and young adult (AYA) generation of T1DM or JIA in the Netherlands (12) provided individual intervention conducted with advice from pediatric and adult medical teams for a year, from setting goals to assessing the effectiveness of efforts to improve care. Patients and the medical team discussed with each other about community barriers and shared their experiences in improving adolescent care. In this one-group pretest-posttest design research revealed that the independent behaviors during consultation, general independence, and self-efficacy increased from before the intervention to 1 year after the intervention.

CHD patients in the United States (24) were provided a personalized education program by pediatric cardiologists to assess and promote the preparation and transition of these patients to age-related adult cardiac care. The TC was developed in January 2016 as a monthly subspecialty pediatric heart clinic. Specifically, during the first visit, the method and importance of transition were explained, the degree of understanding of one's own disease was confirmed, an adult cardiovascular clinic was introduced, and goals for self-management were set. During the second and subsequent visits, re-education about heart disease and resetting of self-management goals were conducted. Interviews with adult CHD nurses were also conducted during the intervention. A TC was set up between pediatric and adult care, and the group was recommended for consultation had a higher follow-up rate than the control group. This non-RCT study showed its effectiveness as a program to promote continuous hospital visits and participation in treatment during the transition period.

## Discussion

### Research Characteristics

The purpose of this systematic review was to provide a multidisciplinary assessment of intervention programs and their effectiveness in supporting the transition of children and adolescents with childhood-onset chronic illness. With the increase in APCCD, support for the transition from pediatric to adult medical care has been emphasized (3), and research reports have been increasing since 2018. DM was the most common disease among the participants of this study, followed by IBD, CF and CHD. In a review of adolescents in transition who require continuous care, the diseases covered were similar to those in this study (26).

Of the 16 research designs, seven were RCTs, six were quasi-experimental studies of one-group pre- and post-test design, and three were non-RCTs, more than half of which were non-RCT research designs. Since the pure experimental study of one-group pre- and post-test design was not a controlled study, it was difficult to determine the extent to which interventions and other factors contributed to self-management, transitional readiness, and improved disease activity. In non-RCT studies, the intervention and control groups were assigned based on the patients' wishes. Therefore, the possibility of selection bias due to the large number of patients or their families who were more interested in the transition from childhood to adulthood, could not be ruled out in the intervention group.

In the bias risk assessment using ROBINS-I, one study had a low overall bias risk, one had serious bias risk, and the rest had moderate bias risk. Bias risks in most studies ranged from moderate to severe, and confounding bias and bias in measuring outcomes were the main factors that underestimated the study quality. The results should, therefore, be interpreted carefully from the study design and bias risk assessment perspective.

The number of RCTs in the four intervention groups was two for IEP, three for GM, two for ALICT, and zero for TC, with no significant differences among the groups other than TC, and the risk of bias in the study design RCTs was Moderate except for one ALICT, which was Low. Based on these results, it was difficult to rigorously estimate which intervention group was more effective.

### Intervention Content and Effect

#### Individual Education Program

The most common transition support intervention program was the IEP. Specifically, the content was to directly provide face-to-face education, discussions, consultations, planning, follow-up, etc. by the supporter, according to the requirements of individual patients. The results of this study and previous studies indicate that there is a wide range of childhood-onset chronic conditions that require transition support (26). In addition, the transition period from childhood to adulthood is a period of rapid physical, psychological, and social maturity. Therefore, it is expected that individual differences will be large in terms of physical and psychological development and changes in social roles. In this way, in order to deal with various diseases and individual situations, it is considered that education and planning are carried out according to individual situations. In many studies, follow-up was conducted not only as a one-time intervention, but also for maintaining the effects.

While many studies included patients and their parents for education, planning and counseling, one study featured interventions regarding the split care of children and parents to facilitate the transition. The purpose of transition support was to support patients' independence and social participation, in addition to a smooth shift from pediatric to adult care (3, 27). Both patients and their parents are the targets of interventions because it is a transitional period from childhood, when parents are responsible for treatment selection and disease management, to adulthood, which aims for patient independence and social participation.

The effects of the IEP included increased knowledge scores regarding illness and health, increased transition preparation scores, increased independence behavior indicators, independence, self-efficacy, improvement of physical symptoms, and shortening the period of consultation from pediatrics to adult specialties. However, one study reported no significant effect on HRQOL and self-management ability. The risk of bias in the study was moderate, and the level of evidence was limited.

#### Group Meeting

GMs for group education and discussions for patients and parents were the second most common support intervention after IEP. Specifically, discussions were held on disease management, medical care after adult transition, and general life including school life and occupation. While most of the studies were regarding patients only, there was one study which included both parents and children. As an independent support for children with chronic pediatric diseases, it is important to provide a place where patients of the same generation, such as peer groups, can share their worries and exchange information (25, 28). Peers become a primary source of support in mid-adolescence, and peer groups are said to participate in self-selection, self-determination, or social participation through exchanging and sharing experiences, information, and ways of thinking (29, 30). Sharing experiences and information with patients of the same generation having the same disease is considered to be effective for future disease management and life planning.

Similar to the IEP, GMs were attended by pediatric and adult health care providers to interact with new care providers. In addition to doctors and nurses, there were also interventions involving professionals from multiple occupations such as social workers, psychologists, and dietitians. As mentioned above, the objectives of transition support included a smooth shift from pediatric to adult care, and support for patient independence and social participation (3, 27). For a smooth shift from pediatric to adult care, both pediatric and adult health care professionals should participate. In addition, we believe that multidisciplinary interventions are being carried out for a wide range of support, including community life along with disease management.

The effects of GMs have been shown to improve knowledge scores, empowerment scores, self-efficacy, transitional ability, disease management status, and physical status. However, one study reported that there was no significant difference in patient activation and quality of life. The risk of bias in the study was moderate, and the level of evidence was limited.

#### Active Learning Using ICT

Under ICT interventions, ICT-based disease management and learning programs, as well as, GMs were held. In addition to recommending records related to disease management using applications, providing information on disease management and lifestyle on the website, and content that allows learning of adult self-management through advanced case studies were set. Alongside one-sided transmission of knowledge and information, active learning was devised by exchanging messages with other target people and holding group meetings using Zoom. In recent years, digital therapy using “therapeutic apps” that can change the behavior of patients when they are not visiting the hospital and their way of thinking by acquiring correct knowledge, has attracted attention as a new treatment method in the United States and Europe (31, 32). With the development of ICT, it has spread rapidly, and medical intervention using smartphone applications that are familiar to university students from adolescence, has shown the possibility of smooth symptom and life management (33, 34). We believe that it is also used in intervention programs related to transition support for the same generation.

As an effect of ICT intervention, an increase in disease self-management ability and self-efficacy was observed, and improvement of patient-led communication was shown by devising interactions such as exchanging messages with other participants. All bias risks were moderate, and the level of evidence was limited. However, the use of ICT and the incorporation of further interaction suggested the possibility of a transition-promoting effect.

#### Transition Clinic

There were two studies in which TCs were set up to assess and promote transition from pediatric to adult care in patients. At the TC, individual education and interviews were conducted by the pediatric and adult health care teams and specialists, such as pediatric cardiologists and adult CHD nurses. Recommendations included assigning doctors with knowledge of transition support to both children and adults, creating teams of specialist nurses, psychological workers, social workers, etc., and establishing organizations for transition outpatients at the American Academy of Pediatrics, the International Society for Pediatric Kidney Disease, etc. (4, 35). Moreover, since many pediatric chronic diseases were highly specific, it was suggested that individual measures should be taken for each disease area, taking into consideration the severity and number of patients (32, 36). Based on these facts, setting up a TC with specialists assigned to each disease area was considered. Although the intervention was shown to be effective in increasing independent behavior, self-efficacy, and continuing hospital visits and promoting participation in treatment during the transition period, the risk of bias was serious and the level of evidence in the study was low.

### Limitations

This study had a few limitations. While we clarified the selection criteria and conducted a comprehensive literature search, there is a possibility that the collection of related literature is insufficient, the search being restricted to those available in English only, and also due to the limited availability of databases. Longitudinal studies without a control group did not show a causal relationship. Moderate risk of bias was also reported in studies with control settings and the results should be interpreted with caution. In addition, it was difficult to accumulate research studies based on a clear definition of transition support intervention programs.

### Suggestions for Research and Practice

As mentioned above, this systematic review lacked the accumulation of studies based on a clear definition of transition support intervention programs. And it was unclear which actions would have a positive effect on which outcomes. The effectiveness of the intervention may also be influenced by the expertise, skills and attitudes of the intervention program provider. Therefore, future studies will require interventions based on a clear definition of transition support intervention programs, evaluation of which actions are effective for which outcomes, and evaluation of program providers.

In some of the studies considered for this systematic review, both pediatric and adult experts were involved in transition support. Specialists in the pediatric and adult fields are medical providers who can provide specialized medical care through different approaches, and it is expected that these two medical providers will provide seamless care (36, 37). In addition, while the number of patients requiring transition support is increasing, the number of medical staff involved in transition support is currently small, and there is an urgent need to expand the education program for transitional medical care for medical staff (35). It is also important to offer personalized support to young people and their parents when assessing the transition readiness, focusing on self-management skills, illness knowledge, and communication with healthcare professionals (38, 39). For these reasons, the future issues in practice are the cooperation system between the pediatric and adult fields, the construction of a seamless medical care system, and the provision and evaluation of educational programs for transitional medical care for the young people, their parents, and medical professionals.

## Conclusion

In this study, we reviewed studies of intervention programs for children and adolescents with childhood onset. The results showed that: (i) DM and IBD were common among the diseases suffered by the study participants, (ii) intervention programs were broadly classified into four categories—IEP, GMs, ALICT and TC, and (iii) education, consultation and planning for disease and disease management through IEPs were common, followed by GMs for group education and discussion. Many studies reported improved knowledge of disease and disease management and improved readiness for transition through the intervention program. Most of the study designs were pre/post comparative studies and RCTs, with the risk of bias being moderate in most of them.

Future challenges include the dissemination of programs with individualized measures for each disease area, as well as the establishment of evaluation and educational methods for program providers.

## Data Availability Statement

The original contributions presented in the study are included in the article/supplementary material, further inquiries can be directed to the corresponding author/s.

## Author Contributions

RW, KS, MY, and YS: conceived or designed the study. RW, KS, MY, AM, and YS: performed research and wrote the paper. RW, KS, AM, and YS: analyzed data. All authors had full access to all data in the study and take responsibility for the integrity of the data and the accuracy of the data analysis. All authors contributed to the article and approved the submitted version.

## Funding

This study was funded by Grant-in-Aid for Scientific Research (Grant Number: 22H00490).

## Conflict of Interest

The authors declare that the research was conducted in the absence of any commercial or financial relationships that could be construed as a potential conflict of interest.

## Publisher's Note

All claims expressed in this article are solely those of the authors and do not necessarily represent those of their affiliated organizations, or those of the publisher, the editors and the reviewers. Any product that may be evaluated in this article, or claim that may be made by its manufacturer, is not guaranteed or endorsed by the publisher.

## Abbreviations

ALICT: Active learning using information and communication technology
APCCD: Adult patients with childhood-onset chronic disease
AYA: Adolescent and young adult
CF: Cystic fibrosis
CFQ-R: Cystic Fibrosis Questionnaire Revised teen/adult version
CHD: Congenital heart disease
CINAHL: Cumulated Index to Nursing and Allied Health Literature
DM: Diabetes mellitus
EA: Esophageal atresia
GM: Group meeting
GSE: General self-efficacy
HRQOL: Health-related quality of life
IBD: Inflammatory bowel disease
IEP: Individual education program
JIA: Juvenile idiopathic arthritis
PAM: Patient activation measure
RCT: Randomized controlled trial
ROBINS-I: Risk of bias in non-randomized studies &#8211
of interventions
SCD: Sickle cell disease
T1DM: Type 1 diabetes mellitus
TC: Transition clinic
TCS: Transition competence scale
TRAQ: Transition readiness assessment questionnaire.
